# The influence of school inclusive education climate on physical education teachers’ inclusive education competency: The mediating role of teachers’ agency

**DOI:** 10.3389/fpsyg.2023.1079853

**Published:** 2023-02-10

**Authors:** Rui Xue, Hongqin Chai, Lei Yao, Wangqian Fu

**Affiliations:** ^1^School of Education, Beijing Sport University, Beijing, China; ^2^Faculty of Education, Beijing Normal University, Beijing, China

**Keywords:** school physical education, inclusive education climate, physical education teacher, inclusive education competency, agency

## Abstract

**Introduction:**

Teachers’ inclusive education competency is the key to the successful implementation of inclusive education. Under the background of China’s vigorous development of inclusive education, the influence mechanism of inclusive education competency of Chinese physical education teachers has not been paid attention to. The present study concentrates on the relationships between school inclusive education climate, physical education teachers’ agency, and inclusive education competency.

**Methods:**

Data were collected from 286 primary and junior high school physical education teachers through nationwide convenience sampling on the Internet in China by completing the School Inclusive Education Climate Scale, Physical Education Teachers’ Agency Scale, PE Teachers’ Inclusive Education Competency Scale.

**Results:**

Results of structural equation modelling revealed that school inclusive education climate had a significant effect on physical education teachers’ agency. School inclusive education climate had a significant influence on physical education teachers’ inclusive education competency. The mediation effect of physical education teachers’ agency on the relationship between school inclusive education climate and inclusive education competency was significant as well.

**Discussion:**

These results demonstrate that school inclusive education climate plays a direct and indirect role in promoting physical education teachers’ inclusive education competency.

## 1. Introduction

Inclusive education is understood as the right of all children to access, present, participate in and successfully attend in their local regular school, which shifts the focus from labels, diagnosis and deficit of some students to quality education for all children (Kielblock and Woodcock, 2023). China’s special cultural background and social system decide that the development of inclusive education in China can not completely copy the western mode of inclusive education (Deng and Su, 2012). At present, the inclusive education in China is still in the exploratory stage. Although inclusive education emphasizes to cater to all students, inclusive education in China lays more emphasis on children with disability and typically developing children (Deng and Zhao, 2019). In June 2020, the Ministry of Education of China issued a guideline on strengthening the work of children with disability attending classes during compulsory education (China, 2020). The “Guideline” includes guidance and opinions on the work of children with disability, indicating that China’s inclusive education is aimed at children with disability, that is, children with disability and typically developing children receive education under the same roof. Currently, in the United States and western countries, teaching students with disability has been studied a lot and there is a whole industry known as Adapted Physical Education (Block, 2016), which is aimed at physical education and sports activities for children with disability (Wu et al., 2022). However, quality physical education advocates meeting the needs of all children, allowing children with disability to enter general physical education classes, participate in physical education activities with typically developing children, enjoy equal rights and opportunities in physical education, and pay maximum attention to individual differences (Pan, 2020). When arranging the content and location of classes, we should not only meet the needs of typically developed children, but also enable children with disability to adapt (Xue et al., 2023). Relevant studies have found that the implementation of quality physical education can improve the active communication behavior of children with disability, stabilize their emotions, improve problem behavior, and improve their social communication and social adaptability (Liu et al., 2012a; Pan et al., 2016; Liu and Si, 2018; Bertills et al., 2021); realize the life care for special students and promote the all-round development of them (Block et al., 2017); and realize educational equity (Wang and Cao, 2014; Haegele et al., 2017). Therefore, improving the quality of physical education can not only promote the physical and mental health growth of children with disability and typically developing children (Qi and Ha, 2012; Liu et al., 2012b), but it can also promote the overall development of inclusive education in China. The quality of education is affected by many factors, including physical factors such as class size and environment (Fletcher et al., 2013) and students’ personal factors (Sanders and Horn, 1998; Savolainen, 2009), but many researchers find that teachers’ competency is the top priority to improve the quality of education (Ye, 1998; Lai, 2019; Zhang and Chen, 2022). Teachers’ competency refers to the basic conditions and abilities of teachers engaged in education and teaching activities (Wang et al., 2018). Under the background of inclusive education, new requirements are put forward for teachers’ competency, including the concept, knowledge, skills, and ability to obtain support of inclusive education, so as to meet the needs of children with disability and typically developing children to receive education under the same roof. It is found that Chinese physical education teachers hold a positive attitude toward quality physical education, but they were concerned about the practical difficulties of teaching children with disability in general physical education classes, the lack of support, and the possible rejection of children with disability by their peers (Wang L. et al., 2015), which indicates that Chinese physical education teachers’ inclusive education competency needs to be improved.

At present, the research on teachers’ inclusive education competency has not been subdivided into specific discipline levels, so the inclusive education competency of physical education teachers has not been paid much attention to in the research, and the research on its influence mechanism is relatively limited. Mining the internal mechanism can provide direction for improving the quality of physical education and provide a basis for promoting the high-quality development of physical education.

Teachers’ competency is affected by the school material environment (Li, 2017), cultural atmosphere (Zhao, 2019), and teachers themselves (Xiang, 2014). In the field of inclusive education, the inclusive competency of inclusive teachers is affected by school section, teaching age (Wang Y. et al., 2015), leadership support, physical environment, inclusive climate, professional support, teaching aids allocation, and other factors (Maeda et al., 2021). Among many factors, the school inclusive climate has attracted much attention. The inclusive climate refers to the relatively stable and lasting environmental characteristics produced by the interactions between teachers and the environment in the process of implementing the inclusive education. There are three views on the elements of the inclusive climate, including the participation of children with disability in school activities, the cooperation between ordinary teachers and special teachers, school principals’ support, and relevant practical activities organized by the school (Schaefer, 2010); second, the mutual help and support among teachers, the support of school leaders and the school’s attention to the academic performance of children with disability (Emam et al., 2022); and third, the support of school principals and the implementation of practical activities (Zhou, 2019). This study adopted Zhou Dan’s view on the constituent elements of the inclusive climate, that is, the school inclusive climate is composed of the support of the school principal and the practical activities carried out by the school. The support of school principals means that the principals themselves recognize inclusive education and provide relevant resource guarantee at the practical level. The school carries out inclusive practice activities, including the planning and deployment of school leaders on the implementation of inclusive education in the whole school, and the activities to promote the development of teachers’ professional ability of inclusive education, such as training related to inclusive education, experience sharing and exchange meetings, etc. A good school climate can improve teachers’ self-efficacy (Malinen and Savolainen, 2016; Ulbricht et al., 2022), create a more inclusively school climate, and improve teachers’ inclusive education efficiency (O’Toole and Burke, 2013; Hosford and O'Sullivan, 2016; Neville et al., 2020). Improving teachers’ attitude toward inclusive education (Fu et al., 2021) is an important variable affecting teachers’ inclusive education competency, therefore, Hypothesis 1 was put forward: the school inclusive education climate has a positive predictive effect on the inclusive education competency of physical education teachers.

Activity theory holds that agency is the ability of human action (Engeström, 2008), including the ability of goal orientation and purposeful action, as well as the ability to use strategies to implement this action (Wertsch, 1985). It is the key for people to turn future ideas into reality (Blackler and Regan, 2009). In the environment of large-scale educational reform, the study found that teachers’ agency plays a substantive and decisive role in the implementation of educational policies (Smith, 2006). Teachers’ agency refers to the ability that teachers show when they make full efforts, actively give full play to their internal potential and initiative in teaching in order to achieve their goals (Valsiner and Van der Veer, 2000), which is the internal driving force of teachers’ development (Dai and Jiang, 2008). The implementation of inclusive education is also a great educational reform for the education system, which has brought new challenges to the education and teaching of physical education teachers. Activity theory holds that external environmental factors need individual agency to affect individual attitudes and behaviors (Engeström, 2008). As an external factor, school inclusive climate also needs individual agency to have practical influence. In the context of inclusive education, the agency of physical education teachers are the psychological characteristics of the positive response to the physical education learning of children with disability and typically developing children, including the sense of teaching efficacy and constructive participation. The sense of teaching efficacy refers to the confidence of physical education teachers that they can successfully carry out quality physical education teaching; constructive participation includes self-regulation and participation in school activities (Zhou, 2019), which refers to the investment degree of teachers in organizing and implementing quality physical education throughout the implementation of quality physical education. Based on this, this study put forward the Hypothesis 2: the school inclusive education climate can positively predict the agency of physical education teachers. Relevant research shows that teachers’ agency can positively predict teachers’ inclusive education competency (Mu et al., 2015; Zhou, 2019). The higher the teacher’s agency is, the more attention is paid to reflecting on teaching strategies, tapping their teaching potential, actively participating in teacher training and professional growth activities, so as to improve their inclusive education competency. Based on this, this study put forward the Hypothesis 3: the school inclusive education climate can indirectly affect their inclusive education competency through the agency of physical education teachers.

To sum up, as an external environmental factor, school climate plays an important role in teachers’ personal and professional development. However, there is no empirical study on how the school inclusive education climate affects the inclusive education competency of physical education teachers. Therefore, this study selected primary and secondary school physical education teachers as the research objects, constructed an intermediary model, and examined the intermediary role of teachers’ agency in the impact of school inclusive education climate on physical education teachers’ inclusive education competency, in order to provide data support for enhancing physical education teachers’ inclusive education competency and promoting the high-quality development of physical education from the perspective of school inclusive education climate.

## 2. Materials and methods

### 2.1. Participants

Participants were physical education teachers from primary and secondary schools chosen through nationwide convenience sampling on the Internet. Our research followed the principles of research ethics and integrity, and all participants provided informed consent on a voluntary basis. The questionnaire was distributed to 317 physical education teachers. Eliminating questionnaires with a large proportion of missing answers and completely duplicated answers, we obtained 286 valid questionnaires representing a response rate of 90.2%. Table 1 displays the demographics of the participants.

**Table 1:** Demographics of the participants (*N* = 286).

Characteristic	Total	Percentage
Gender		
Male	228	79.7
Female	58	20.3
School district		
Northeastern	78	27.3
Eastern	88	30.8
Central	53	18.5
Western	67	23.4
Education background		
Master degree or above	40	14.0
Bachelor degree	242	84.6
College degree or below	4	1.4
Years of teaching		
Under 5 years	142	49.7
6 ~ 15 years	80	28.0
Above 16 years	64	22.4
School level		
Primary	82	28.7
Junior	90	31.5
High	114	39.9

### 2.2. Measures

#### 2.2.1. School inclusive education climate

The school inclusive education climate was measured the School Inclusive Climate Scale developed by Zhou (2019). The scale consisted of nine items and was divided into two subscales: school principals’ support and participation in relative activities. A 5-point Likert scale was used from 1 (very disagree) to (very agree), and the higher the score, the better the school IE climate. In the present study, the Cronbach’s *α* coefficients of each subscale were 0.949 and 0.973, and the Cronbach’s *α* coefficient of the whole scale was 0.974, demonstrating a good reliability of the scale. Furthermore, the goodness of fit (χ^2^/df = 1.592, GFI = 0.956, AGFI = 0.895, RMSEA = 0.065) showed a good validity.

#### 2.2.2. Physical education teachers’ agency

This study adopted a self-reported questionnaire of teachers’ agency developed by Zhou (2019) to investigate physical education teachers’ agency, which included 16 items. The scale consisted of two dimensions: teaching efficacy and constructive participation. This study used a 5-point Likert scale from 1 (very disagree) to (very agree) and a higher score suggested a high level of physical education teachers’ agency. The Cronbach’s *α* coefficients were 0.963 and 0.964, respectively, for each dimension, and the Cronbach’s *α* coefficient of the total questionnaire was 0.981, showing a good reliability. Meanwhile, the scale had a good validity (χ^2^/df = 1.899, GFI = 0.951, AGFI = 0.889, RMSEA = 0.077) in the present study.

#### 2.2.3. Physical education teachers’ inclusive education competency

The Inclusive Education Teacher’s Professional Capacities Scale developed by Wang L. et al. (2015) was used to assess physical education teachers’ inclusive education competency, which was a 28-item with four subscales (professional attitude, professional knowledge, professional skills, and the ability to obtain support). The scale adopted a 5-point Likert scale from 1 (very disagree) to (very agree), and a high score indicated a high level of inclusive education competency of physical education teachers. The Cronbach’s *α* coefficients were 0.931, 0.951, 0.958, and 0.952 respectively, and the Cronbach’s *α* coefficient of the total scale was 0.968, showing a good reliability. The CFA was further performed and the fitting indexes were χ^2^/df = 1.416, GFI = 0.968, AGFI = 0.922, RMSEA = 0.051, indicating that the reliability of the scale was good.

### 2.3. Data analysis

In this study, the data were obtained by issuing the questionnaire, carefully screening the questionnaire, and eliminating the extreme value and abnormal value questionnaire. SPSS 26.0 software was used to test the reliability, validity, and correlation analysis of the data. Using Amos 26.0 software, confirmatory factor analysis on the data and constructs structural equation model could be carried out.

## 3. Results

### 3.1. Common method bias investigation

In order to control the potential common method bias, firstly, the Harman single-factor test was used to test the common method bias. Exploratory factor analysis was conducted on all items of the questionnaire, and common factors were extracted by principal component analysis. The results showed that the variance explanation rate of the first factor without rotation was 28.157%, less than 40%. In summary, there was no serious common method bias in the data of this study.

### 3.2. Correlational analysis of school inclusive education climate, physical education teachers’ agency, and inclusive education competency

Table 2 presented the means (M) and standard deviations (SD) of all dimensions, and summarized the correlations between school inclusive education climate, physical education teachers’ agency, and inclusive education competency. Spearman’s correlation showed that there was a significant positive correlation between school inclusive education climate and physical education teachers’ agency (*p* < 0.01), and the correlation coefficient r ranged from 0.663 to 0.715; there was a significant positive correlation between school inclusive education climate and inclusive education competency (*p* < 0.01), and the correlation coefficient r ranged from 0.392 to 0.615; there was a significant positive correlation between physical education teachers’ agency and inclusive education competency (*p* < 0.01), and the correlation coefficient r ranged from 0.555 to 0.646.

**Table 2:** Descriptive statistics and Spearman’s correlation coefficients.

	School inclusive education climate		Physical education teachers’ agency		Inclusive education competency			
	(1)	(2)	(3)	(4)	(5)	(6)	(7)	(8)
School principal support (1)	—							
Participation in school-wide inclusive practices (2)	0.879^**^	—						
Teaching efficacy (3)	0.715^**^	0.680^**^	—					
Constructive participation (4)	0.686^**^	0.663^**^	0.945^**^	—				
Professional attitude (5)	0.409^**^	0.392^**^	0.646^**^	0.630^**^	—			
Professional knowledge (6)	0.521^**^	0.604^**^	0.580^**^	0.555^**^	0.530^**^	—		
Professional skills (7)	0.496^**^	0.537^**^	0.624^**^	0.603^**^	0.481^**^	0.765^**^	—	
The ability to obtain support (8)	0.571^**^	0.615^**^	0.644^**^	0.620^**^	0.525^**^	0.698^**^	0.850^**^	—
Mean	3.86	3.81	4.06	4.14	4.08	3.41	3.73	3.64
SD	1.00	1.06	0.80	0.77	0.79	0.97	0.89	0.90

^**^p < 0.01.

### 3.3. Relationships between school inclusive education climate, physical education teachers’ agency, and inclusive education competency

Based on the correlation analysis of the above dimensions, it was found that there was a significant correlation between school inclusive education climate, physical education teachers’ agency, and inclusive education competency. To test the mediating effect of physical education teachers’ agency in the relationship between school inclusive education climate and inclusive education competency, a structural equation modelling was constructed. Having set up the validity of the measurement model, the model was tested. Results revealed that the model fit well. Table 3 presents the model fitting indexes.

**Table 3:** Evaluation of model fit.

Fitting index	Criterion	Value
χ^2^/df	<3	1.689
GFI	>0.9	0.967
AGFI	>0.9	0.908
NFI	>0.9	0.982
IFI	>0.9	0.992
TLI	>0.9	0.984
CFI	>0.9	0.992
RMSEA	<0.08	0.070

**Figure 1:**
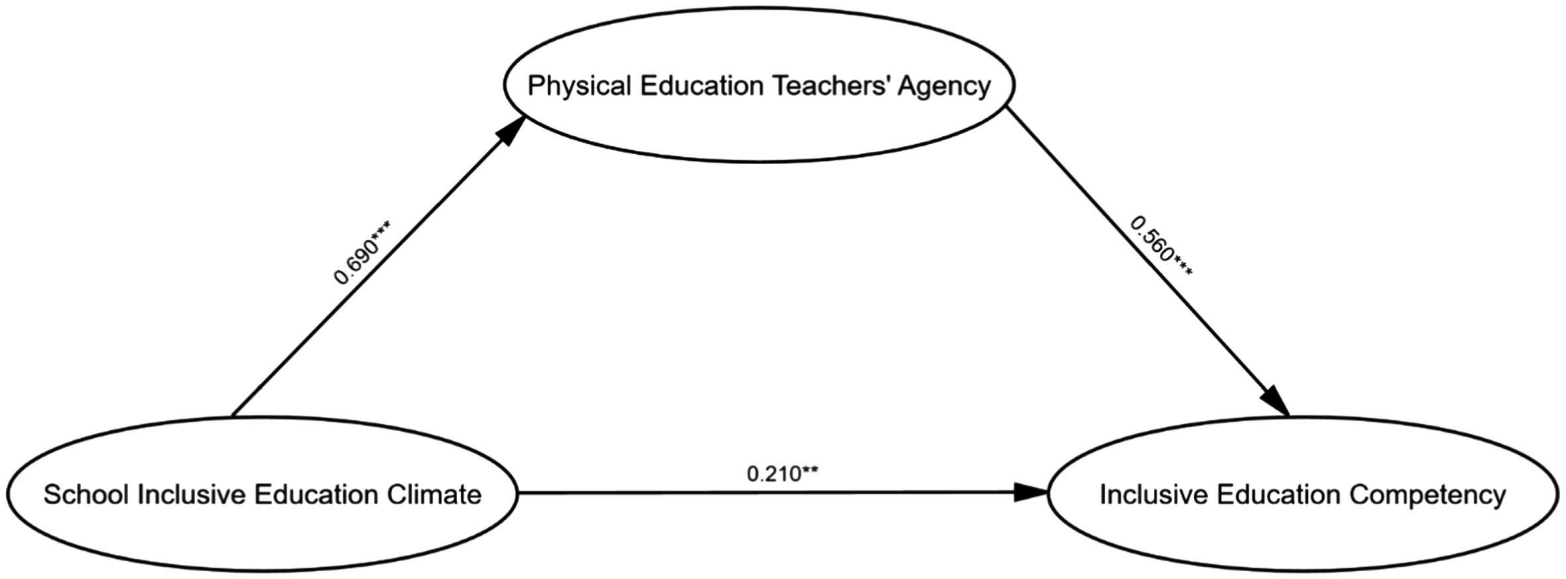
The mediation model showing impacts of school inclusive education climate on inclusion education competency through physical education teachers’ agency.

Figure 1 presents the standardized path coefficients among school inclusive education climate, physical education teachers’ agency, and inclusive education competency. As shown in Figure 1, the standardized path coefficient of school inclusive education climate on physical education teachers’ agency was 0.690 (*p*<0.001); the standardized path coefficient of physical education teachers’ agency on inclusive education competency was 0.560 (*p*<0.001); the standardized path coefficient of school inclusive education climate on inclusive education competency was 0.210 (*p*<0.01). In summary, the results supported Hypotheses 1 and 2, manifesting that school inclusive education climate had a significant direct impact on both physical education teachers’ agency and inclusive education competency.

Bias-corrected bootstrap analysis (bootstrap = 1,000) was applied to test the mediating effect of physical education teachers’ agency because bootstrap analysis had statistic power on testing mediation effects in research with small to moderate sample sizes. The mean path coefficients of the mediation effect and confidence intervals (CIs) were calculated in this analysis. The results showed that 0 did not fall within the range between the lower CI and the upper CI, which indicated that the mediation effect was significant. The direct effect value of the influence of school inclusive education climate on inclusive education competency was 0.279, and the indirect effect value of the influence of school inclusive education climate on inclusive education competency through physical education teachers’ agency was 0.381. To sum up, the results support Hypothesis 3. Table 4 displays detailed statistic information and CIs at the 95% level.

**Table 4:** Direct and indirect effects of school inclusive education climate on inclusive education competency *via* physical education teachers’ agency.

SIEC – IEC	Path	Value	Bootstrapping				
			Percentile 95% CI		Bias-corrected percentile 95% CI		Two tailed significance
Direct effects	SIEC→IEC	0.279	0.154	0.438	0.159	0.449	0.00(^**^)
Indirect effects	SIEC→PETA→IEC	0.381	0.241	0.521	0.261	0.561	0.00(^**^)

SIEC, school inclusive education climate; IEC, inclusive education competency; PETA, physical education teachers’ agency; CI, confidence intervals. *N* = 286; bootstrapping = 1,000; ^**^p<0.01.

## 4. Discussion

### 4.1. School inclusive education climate had a positive impact on physical education teachers’ inclusive education competency

This study found that the school inclusive education climate can positively predict the inclusive education competency of physical education teachers, and Hypothesis 1 had been verified. Wang Y. et al. (2020) investigated regular class teachers in primary and secondary schools and found that the school inclusive climate had a positive and direct impact on the inclusive education competency of regular class teachers, which was consistent with the results of this study. Roosevelt’s theory of the cultural essence of human development points out that cultural ecology is an important factor affecting human development, and human development is a process of “transformation in the process of participation” in and cultural process (Huang, 2009). Schools constitute the cultural ecological environment of teachers’ social work, so the research on teachers’ professional development and personal professional quality is inseparable from the investigation of teachers’ environment. The school values and school running ideas can edify, demonstrate, standardize, and regulate the cultivation and formation of teachers’ professional quality, while teachers can understand, master, and recognize the expectations and norms of school culture (Ru, 2012). As the core leader of the school, the principal has solid knowledge related to inclusive education and advanced inclusive education concept, can set an example for teachers to implement inclusive education, and promote teachers to form a sense of identity with the school running concept and teaching practice of inclusive education.

Meanwhile, the institutional culture regulates and adjusts teachers’ behavior and guides school teachers to develop in the direction guided by the cultural objectives of the school system (Ru, 2012). The school takes the development of teachers’ inclusive education competency as the key work content, and formulates some training practice activities and learning systems for teachers, which is conducive to enriching the reserves of teachers’ inclusive education professional knowledge and skills. The school provides sufficient material and spiritual support for the development of inclusive education, which is also conducive to breaking the obstacles of teachers in the process of implementing inclusive education and strengthening teachers’ ability to obtain school, parents, and social support. In addition, the social cultural theory (SCT) developed from Vygotsky’s “culture history theory” emphasizes paying attention to social culture and individual psychology. It believes that human cognitive development is an internalization process in which individuals integrate favorable factors in the external environment into their own cognitive system (Li and Li, 2021). According to this theory, teacher learning is one of the types of teacher cognitive development, and the improvement of teachers’ inclusive education competency is an important result of teachers’ learning. The positive effects brought by the school inclusive education climate will help to improve teachers’ inclusive education competency. Therefore, a harmonious school inclusive education climate can improve the inclusive education competency of physical education teachers.

### 4.2. The mediating effect of physical education teachers’ agency

This study found that there was a significant positive correlation between school inclusive education climate, physical education teachers’ agency, and teachers’ inclusive education competency, and school inclusive education climate indirectly affected their inclusive education competency through physical education teachers’ agency. Hypotheses 2 and 3 had been verified. A good school inclusive education climate is conducive to stimulating the endogenous motivation of PE teachers, giving play to their subjective initiative, studying and exploring actively, and improving their inclusive education competency, which is consistent with the previous research results (Wang Y. et al., 2020). First of all, an inclusive school education climate helps to improve the agency of physical education teachers. Previous studies have found that the school climate perceived by teachers is significantly positively correlated with the self-efficacy in teachers’ agency (Wang Z. et al., 2020), and the school climate including organizational support and colleague support can positively predict teachers’ agency (He et al., 2018; Qi et al., 2020). The supportive school inclusive education climate can provide support for teachers to improve teaching level and teaching methods, stimulate teachers’ agency, and enthusiasm to participate in the implementation and development of inclusive education, and improve the level of agency.

Secondly, physical education teachers’ agency helps to improve their inclusive education competency. Xu and Long (2020) pointed out from the ecological perspective of applied linguistics that resources in the environment provide opportunities for individual actions, but the occurrence and realization of actions must also rely on individual agency, so the personal growth of physical education teachers also needs the exertion of their subjective agency. Previous studies have found that teachers have high subjective agency, which can promote them to actively acquire and create learning opportunities, increase their reserves of professional knowledge and skills, and then improve their competency (Xu and Long, 2020).

In addition, according to the life course theory, teachers play their agency in the school environment and build their own professional development process by making “choices” and taking “actions” (Elder et al., 2003). The improvement of teachers’ inclusive education competency occurs in their professional development process and is the result of the interaction between the school inclusive education climate and the agency of physical education teachers. Therefore, the stronger the school inclusive education climate perceived by physical education teachers and the higher the agency of teachers, then teachers may take more actions to learn relevant knowledge and skills of inclusive education and obtain support for inclusive education, so as to improve their inclusive education competency.

## 5. Limitation and future research

There were still some limitations in this study. First, the sample distribution was uneven. Although there were participants from many provinces in China, most of them are from the north area of China. Future research should increase the number of participants and pursue the relative balance of the number of subjects in each region. Second, this study adopted a cross-sectional study, and the school inclusive education climate, teachers’ agency, and teachers’ inclusive education competency have dynamic characteristics. Future studies might benefit from adopting longitudinal study in the research methods, so as to better explore the influence mechanism of physical education teachers’ inclusive education competency. Third, this study only discussed the principals’ supportive philosophy and practical measures for inclusive education in the school climate, such as encouraging schools to carry out inclusive education and providing relevant training courses for teachers. However, it was not discussed whether there are other factors in the school climate that affect the physical education teachers’ inclusive education competency, whether there are factors and reasons that exclude inclusive education in the school environment, which can be further supplemented and explored in future research. And the results in this study was limited by cultural climate and educational development. Inclusive education in China focuses more on children with disability, therefore, attention to underrepresented children, international children, LGBQT+ children, etc. should be further paid to in future research.

## 6. Implications

There was a significant correlation among the three variables: school inclusive education climate, physical education teachers’ agency, and physical education teachers’ inclusive education competency. The school inclusive education climate positively predicts the agency of physical education teachers and inclusive education competency. The school inclusive education climate can affect the inclusive education competency of physical education teachers through the mediating role of agency. The better the school inclusive education climate, the more it can improve the agency of physical education teachers. The stronger the agency of physical education teachers for quality physical education, the higher their inclusive education competency. Teachers’ inclusive education competency is affected by teachers’ internal and external factors (Wu, 2017; Wang, 2021). The results of this study provide two potential measures to improve the inclusive education competency of primary and secondary school physical education teachers.

First, we should build a good school inclusive education climate to enhance the impact of external factors on physical education teachers’ inclusive education competency. On the one hand, strengthening the training of principals in inclusive education, constantly updating and strengthening the principals’ inclusive education ideas and ideas, so as to strengthen the principals’ support for the inclusive education of the school; On the other hand, the school should actively organize training activities related to inclusive education, such as organizing in-school inclusive education learning seminars, visiting and learning by expatriate teachers, etc.

Second, improve the agency of physical education teachers to increase the impact of internal factors on physical education teachers’ inclusive education competency. Some studies have shown that teachers’ agency is affected by factors such as self-orientation, external forces, collective cooperation, school power relations, social roles in schools, and so on (Zhang and Shen, 2012; Kline et al., 2013). Therefore, when organizing training related to inclusive education, due to the stronger practicality and operability of physical education, the school should carry out targeted special training for physical education teachers to improve their sense of mission and responsibility. At the same time, all schools should actively cooperate with special schools to create a shared and harmonious school climate and improve the agency of physical education teachers. Finally, we should promote the reform and transformation of school leadership and give physical education teachers the opportunity to participate in the development of school inclusive education.

## 7. Conclusion

Inclusive education helps children with disability and typically developing children grow up together, which reflects humanistic care. This study found that inclusive school climate had a positive predictive effect on physical education teachers’ inclusive education competency, and supportive inclusive education climate was conducive to the development of physical education teachers’ professional ability and promote the development of quality physical education. In addition, the study has tested the mediating effect of physical education teachers’ agency in the relationship between inclusive education climate and inclusive education competency. Teachers’ agency is essential in transforming the external environment support into the improvement of individual internal competency, and hence teachers’ agency plays a mediating role. This study proposed that we should improve the inclusive education competency of physical education teachers from two aspects: improving the climate of inclusive education in schools and enhancing the agency of teachers.

## Data availability statement

The raw data supporting the conclusions of this article will be made available by the authors, without undue reservation.

## Ethics statement

The studies involving human participants were reviewed and approved by the Ethics Committee of Beijing Sport University on October 25, 2022. The patients/participants provided their written informed consent to participate in this study.

## Author contributions

RX: conceptualization, methodology, and writing—original draft preparation. HC: investigation, formal analysis, resources, and data. LY: review and editing. WF: supervision. All authors contributed to the article and approved the submitted version.

## Funding

This work was supported by the Special Fund for Basic Scientific Research Business Expenses of Central Universities in 2021 under grant number 2021NTSS34 and the 67th Batch of General Support from China Postdoctoral Science Foundation under grant number 2020 M670186.

## Conflict of interest

The authors declare that the research was conducted in the absence of any commercial or financial relationships that could be construed as a potential conflict of interest.

## Publisher’s note

All claims expressed in this article are solely those of the authors and do not necessarily represent those of their affiliated organizations, or those of the publisher, the editors and the reviewers. Any product that may be evaluated in this article, or claim that may be made by its manufacturer, is not guaranteed or endorsed by the publisher.
